# The general practitioner and mental health problems: challenges and strategies for medical education

**DOI:** 10.1590/S1516-31802005000200008

**Published:** 2005-03-02

**Authors:** Dinarte Alexandre Ballester, Ana Paula Filippon, Carla Braga, Sérgio Baxter Andreoli

**Keywords:** Medical education, Continuing education, Primary health care, Mental disorders, Qualitative research, Educação médica, Educação continuada, Cuidados primários de saúde, Transtornos mentais, Pesquisa qualitativa

## Abstract

**CONTEXT AND OBJECTIVE:**

Within the context of primary health care and mental disorders, our aim was to study the opinions of general practitioners regarding attendance of people with mental health problems.

**DESIGN AND SETTING:**

Qualitative focal group study among primary care services in the cities of Porto Alegre and Parobé, State of Rio Grande do Sul.

**METHODS:**

A deliberately selected sample of 41 general practitioners who were working in basic health services met in focal groups. Two videos were presented, which simulated consultations for patients with depression and psychoses. The discussions about the identification and handling of mental health problems were recorded and assessed via content analysis.

**RESULTS:**

The opinions related to the difficulties of diagnosing and treating mental problems, the involvement of relatives in caring for patients, the difficulty of compliance with the treatment, the uncertainty experienced by physicians and the difficulty of referring patients to specialized services.

**CONCLUSIONS:**

The general practitioners indicated that they perceived the mental health problems among their clientele, but the diagnosis and treatment of these problems are still seen as a task for specialists. The challenge of continuing education on mental health requires methods of interactive and critical teaching, such as the problem-based approach.

## RESUMO

**CONTEXTO E OBJETIVO:**

No contexto de cuidados primários de saúde e transtornos mentais, o objetivo foi conhecer as opiniões de médicos generalistas sobre o atendimento de pessoas com problemas mentais.

**TIPO DE ESTUDO E LOCAL:**

Estudo qualitativo, por grupos focais, nos Serviços Básicos de Saúde em Porto Alegre e Parobé, Rio Grande do Sul.

**MÉTODOS:**

Um grupo de 41 médicos que trabalham em cuidados primários de saúde foi escolhido de modo intencional e reunido em grupos focais. Foram apresentados a eles dois vídeos, que simulavam o atendimento de pacientes com depressão e psicose. Discussões sobre a identificação e o manejo dos problemas mentais foram gravadas e descritas pelo método de análise de conteúdo.

**RESULTADOS:**

As principais opiniões dos médicos referiram-se às dificuldades para diagnosticar e tratar os problemas mentais, o envolvimento dos familiares no cuidado dos pacientes, a dificuldade de adesão ao tratamento, as incertezas experimentadas pelos médicos e as dificuldades para referenciar os pacientes aos serviços especializados.

**CONCLUSÕES:**

Os médicos generalistas percebem os problemas mentais na sua clientela, mas consideram que o diagnóstico e tratamento destes problemas é tarefa do especialista. O desafio da educação continuada em saúde mental requer métodos de ensino interativos e críticos, como a abordagem de solução de problemas.

## INTRODUCTION

Mental disorders have high rates of prevalence in the general population and represent a significant potential demand for basic healthcare services. It is estimated that 25 to 30% of consultations in general outpatient services are related to such problems.^1–4^ However, general practitioners find it difficult to diagnose and apply effective treatments to mental disorders.^5^ Furthermore, they are usually very busy, and also skeptical about their role in mental health assistance.^6^

The difficulty in diagnosing is thought to be not only due to lack of time for seeing such patients, but also because specific skills are needed for practicing good outpatient medical care.^7^ Physicians are trained to diagnose according to illness category but, with regard to mental problems, they have difficulties in using psychopathological language. One important challenge in diagnosing mental disorders is the imprecise biological basis for them, although recent advances in neurosciences have been bringing in meaningful contributions. Classification systems for mental disorders, such as the Diagnostic and Statistical Manual of Mental Disorders and the Classification of Mental and Behavioral Disorders, primary care version, have also contributed towards an integrated evaluation of patients, but they may be not so accessible for the general practitioner.^8,9^

Treatment is another challenge that needs to be faced from various angles: development of communication skills, consideration of which interventions are most available and most appropriate among a broad range of existing treatments, prescription of drugs according to the best scientifi c evidence, consideration for patients’ preferences and, finally, recognition of the inevitable limitations.^10,11^ Consequently, as well as the need for new knowledge, a change of attitude is required, and this will have an influence on the learning process, as well as on the contact with the patients.^12,13^

A thorough review of the literature on mental health and primary healthcare reveals that psychiatrists and general practitioners do not always agree in their opinions on the most important topics to be discussed within continuing education. Furthermore, programs focused exclusively on the diagnosis and use of medicines may disregard the main goal for clinical practice in primary care: the capacity to develop and maintain a relationship with patients who have complex problems, with the aim of facilitating attendance and treatment effectiveness. Evaluation of studies on the teaching of psychiatry in primary care point towards the same fact: to enable acquisition of new knowledge and skills, an open attitude and engagement are needed in relation to the attendance of patients with mental problems. Programs based exclusively on knowledge have had little or no impact on changes in attitude. It is necessary to include opportunities for general practitioners to discuss their perceptions of psychiatry, mental problems and the attention given to these problems in the health system.^14^

The present study was developed within a training program for general practitioners who work in primary care services, with the objective of ascertaining these professionals’ opinions about attendance for people with mental problems, so that educational techniques regarding mental health that take these professionals’ learning characteristics into account can be developed.

## POPULATION AND METHODS

The study followed a qualitative approach towards evaluating the opinions of a group of 41 physicians (internists, pediatricians, gynecologists-obstetricians, occupational health physicians and family and community physicians) who were working in primary care services in the cities of Porto Alegre and Parobé, State of Rio Grande do Sul. The choice of population was deliberate: the participants were resident physicians in family and community medicine from Murialdo Health Center, State Health Department of Rio Grande do Sul, and from the outpatient department of a shoe factory.

The technique for data collection consisted of focal groups. The participants were gathered in three groups: two of ten individuals each and a third group of 21 individuals. The meetings of the groups were part of a training program in mental health, and they were preceded by another three meetings that had the objective of examining case studies regarding seven mental health problems (depression, sleeping problems, alcohol and drug problems, anxiety, dementia and unexplained somatic complaints). Two videos produced especially for this study and were presented in the focal groups, which simulated consultations for patients with depression and psychoses that lasted for 8 and 5 minutes, respectively. Initially, some questions were put to the groups, to stimulate the participants to express their opinions about the identification and management of the problems presented in the videos. The conversations were recorded and transcribed. Each session lasted around 60 minutes, with active and different manifestations from the participants. The strategy of using focal groups had the objective of gathering opinions that could help in identifying barriers against change and in planning interventions.^15–17^

The videos followed a schedule in relation to diagnostic aspects (present complaints, diagnostic criteria and differential diagnosis) and treatment (essential information for the patient and relatives, recommendations for the patient and relatives, medication and the advice to seek a specialist), based on the Classification of Mental and Behavioral Disorders, primary care version.^18^

The transcribed texts from the group recordings were assessed via the content analysis technique.^19^ The analysis categories were built up from the units of meaning that were identified and the observed material. Qualitative research evidence may be very useful in health service evaluation and help in comprehending human behavior in its social context.^20^

## RESULTS

The reports from the groups showed several indicators of physicians’ opinions, and analysis of the content of the texts allowed the following categories to be drawn up:

### 1. Opinions about the diagnosis of the problem

*"I started looking differently at those patients who are always in the health center and asking them other kinds of questions".*

*"General practitioners have a harder investigative task. Patients may arrive at the health center with a lot of complaints, and we are the ones to raise the hypothesis of a psychiatric illness".*

The physicians agreed that mental problems are very common in basic healthcare services and considered that they could take care of these people if they received the necessary training. They agreed that, at medical school, the teaching is directed towards the training of specialists, and that is why they tended to think that long consultations are needed in relation to mental problems, i.e. "psychiatrist's time". They considered that complementary tests are sometimes indispensable for the diagnosis. They found it necessary to fit the theoretical knowledge in with the practice, and to ask questions such as: How should the normal be differentiated from the pathological? What are the clues for the diagnosis? How should diagnostic categories be used? How should suicide risk be identified risk? How should organic causes be identified?

### 2. Opinions about the handling of the problem

*"In general, we leave college knowing the theory. What we lack is hands-on practice."*

*"What we see the most is the prescription of benzodiazepine to treat sadness rather than depression… It's easier, so that the patient doesn't talk during the consultation, which would make it longer."*

The groups felt more confident about dealing with depressive patients, but expressed their inexperience regarding severe mental problems such as psychoses. Some characteristics that would making attending psychotic patients more difficult were the long term evolution, the risk of aggression and the longer time required for attending to them.

The physicians considered that there were problems in continuing the treatment between crises, because of the difficulty in obtaining compliance from the patients. They recognized the importance of the physician's attitude in linking their training with the continuity of the treatment, i.e. the way the physician talks with patients: how he advises them and explains about the illness. They became aware that they had doubts about how to handle critical situations, such as suicide, and considered that the physician, the healthcare team and the family's conduct has an impact on these critical events. Home visits were seen as a strategy for improving the relationship between the healthcare team and the patient.

In the groups’ opinions, the therapeutic arsenal includes medicines, psychotherapy, family support and activities. Some dilemmas came up: When should one prescribe a drug? How should the medication be adjusted? When should one indicate psychotherapy? How should emergencies be dealt with? When should one indicate hospitalization? How should other support services for the patient be used? How should the continuity of the treatment be maintained? Faced with these dilemmas, the physicians expressed the fear of having inadequate conduct, as well as the surprise that it might be possible to solve the problem. They admitted that it was possible to treat mental health problems in primary care, recognizing the importance of the setting for handling the patient. They questioned the indiscriminate use of medicines, especially benzodiazepine, and believed that the formation of a therapeutic link is something that requires a long time.

### 3. Effect of the family on the patient

*"The families that we attend are very problematic. They have thousands of problems and get depressed."*

*"Conducting a dialog is difficult for those who don't have any training. Uncommunicative patients don't display any logic: What can I do to talk to them? In such cases, the relationship is physician-family-patient."*

*"…sometimes, the patient must be restrained, or the family may be able to take care of him/her at home, and we don't know how to deal with that."*

In the groups’ opinions, there was a need for involvement by the patients’ families as allies in working towards success for the treatment. However, at the same time, they noticed that some families were reckless towards the patient's care. They considered that physicians must exercise a responsibility regarding the relatives of people with mental problems.

### 4. Patients’ feelings perceived by the physician

*"If the general practitioner gives the psychiatric diagnosis, the patient doesn't accept it, but when the specialist says so, it's a law."*

*"When the patient goes to the psychiatrist he already has the notion that he has a psychiatric illness; he's already accepted that."*

The physicians noticed the difficulty that patients had in accepting the diagnosis and complying with the treatment. They considered that the teaching model used for medicine, which is directed towards the training of specialists, is exposed to discredit from patients in relation to general practitioners, and that this generates insecurity for clinical practice.

### 5. Feelings provoked in the physician by the patient

*"The psychiatric patient causes fear in the physician, or the fear of not knowing how to deal with the situation." "They have a lot of problems and get depressed. I think, if it was me, I'd already have killed myself. It's complicated to deal with that."*

The groups expressed a variety of feelings: inexperience, insecurity and uncertainty, especially when attending psychoses and critical situations, while also varying as far as the satisfaction of being able to deal with mental problems. Other feelings were: fear related to "madness"; fear of scaring the patient away with the diagnosis of "madness"; fear of being attacked by psychotic patients; and difficulty in establishing a link with the patient.

### 6. Difficulties with the organization of the services

*"The difficulty with these patients is that we don't get referrals: we don't get a psychiatrist."*

*"In emergencies, patients arrive in a situation of crisis. When we don't get a referral, we end up sedating them for two or three days."*

The physicians recognized the need for institutional resources such as health services and social assistance for attending to individuals with mental problems. They considered that there were not enough of these services yet, particularly for attending to psychotic patients, but they foresaw a tendency towards improvement. They found difficulties in referring patients with mental disorders to the specialized services, and concluded that the disorganization of the health system generates a lack of continuity and a large demand for attendance.

## DISCUSSION

A fundamental component in the formation of attitudes is the affective dimension. The cognitive and behavioral aspects, i.e. the elaboration of ideas and the actions derived from them, are intrinsically related to the feelings that go through thoughts and conduct. From this complex relationship of cognition-affection-conduct, a more or less fertile soil for learning will result.^21^ In the present study, fear took the form of uncertainty, insecurity, inexperience and vulnerability of the other person. This reaction may originate from the stigma of mental illnesses, from which physicians are not immune, and this may contribute towards the rejection of "madness" in its various meanings and the consequent separation of "mad" individuals. Consequently, the acquisition of knowledge and formation of attitudes in relation to patients with mental problems may be subject to the feelings experienced by the physician in his work, and to his imagination regarding mental illness, thereby either facilitating or causing difficulty to the learning process.

The participants suggested that the mental health training received at medical schools had led to rather limited results. Even though the disciplines of psychiatry and medical psychology are included in the curriculum, difficulties arise in professional practice when it is necessary to identify and deal with mental problems, from the least to the most severe cases. Over the last 50 years, several studies have pointed in the same direction: it is necessary to improve medical teaching in relation to mental health problems. The doubt is still what to do to accomplish this enormous task. The educational requirements for facing the challenges of diagnosing and dealing with mental problems demand an attentive and critical attitude. This process must start at undergraduate level and continue throughout professional development. For reaching this goal, problem-based learning and interaction in small groups have been shown to be superior to the traditional teaching models, which are limited to exposure to information.^22^

These innovations in medical teaching are student-centered and based on priorities because, in the education of adults, the learning trigger is the ability to overcome challenges and solve problems. Learning through problem solving also leads students to experience uncertainties, for instance about what and how to study. In health-care attendance, the professional may face uncertainty when choosing the best way to act. This condition of uncertainty must lead to reflection and a search for knowledge to make decisions. The capacity to bear up to uncertainties in the comprehension of the phenomena and the search for solutions, which are very important in relation to mental health clinical care, may constitute a meaningful contribution towards the integrated training of the physician.^23–25^

The problem-based approach used by general practitioners has demonstrated its effectiveness and acceptability in the treatment of mental disorders in primary care.^26^ In this sense, the possibility of working in primary care with the support of specialists through psychiatric consultations may also be an important tool for continuing education.^27^

The group discussions raised several themes that were not discussed in the training program, considering the general physician's point of view: psychiatric emergencies, mental problems associated with other physical conditions and communication problems in the physician-patient relationship. Other themes were discussed while studying the cases, but doubts about treatments with psychoactive drugs and psychotherapies still remained, as well as the difficulties of knowing how to refer patients to the health system.

Educational evaluation studies have shown that continuing extensive educational programs with reinforcement and periodic evaluations are necessary to enable changes in clinical practice and patient evolution to take place. The tendency is that interactive programs that are relevant for the context may develop the knowledge, skills and attitudes of the general practitioner when attending to people with mental disorders.^28^

## CONCLUSIONS

The opinions of the general practitioners indicate that they perceive the mental health problems among their clientele, but as something far from their intervention. The diagnosis and treatment of mental problems are still seen as a task for specialists, since this may demand lengthy time and sophistication. Nevertheless, in their daily clinical practice, they face having to attend to people with mental disorders, possibly even in a situation of crisis, and they do their best to find solutions among the various doubts and dilemmas.

The challenge of continuing education for doctors in relation to mental health requires methods for interactive and critical teaching, such as the problem-based approach. Thus, new educational developments that are appropriate for professional needs and the real problems of these doctors’ communities are still needed for improving primary mental healthcare.
